# Supplementary material for: A nuclear clock synchronized to 229Th
Source: Nature. 2026 Oct 7;658(8137):903–8. doi: 10.1038/s41586-026-11122-1 (PMC13686941; doi:10.1038/s41586-026-11122-1)
Supplement: Supplementary file 1 [file 41586_2026_11122_MOESM1_ESM.pdf]

nature portfolio

Peer Review File

A nuclear clock synchronized to ^{229}Th

Corresponding Author: Professor Shiqian Ding

Any redactions in this file are there to maintain patient confidentiality, the confidentiality of unpublished data, or to remove third-party material.

This file contains all reviewer reports in order by version, followed by all author rebuttals in order by version.

Version 0:

Reviewer comments:

Referee #1

(Remarks to the Author)
The manuscript "A nuclear clock based on 229Th" by B. Huang et al. presents the first demonstration of a solid-state nuclear clock operation, in which the frequency of the 148-nm CW laser is stabilized to the thorium-229 nuclear transition. The error signal for the feedback loop was obtained via lock-in detection of the absorption signal. Clock operations using combinations of two home-grown Th:CaF2 crystals and two VUV photon detectors (PMT and Phototube) were demonstrated. A clock comparison between the solid-state nuclear clock and a highly stable laser source stabilized to a cryogenic silicon cavity was also demonstrated. Frequency reproducibility among the home-grown crystals has been confirmed at the 10^-13 level, which should provide important insights into future crystal fabrication to improve clock performance. The manuscript is well written, technically sound, and fully convincing. The achievements are worthy of publication in Nature.

However, I have significant concerns regarding the data analysis of the clock's instability. Therefore, I do not recommend the publication of the manuscript in its current form. Below, the authors will find all my concerns that would need to be considered.

1.Figure 2.
-Fig. 2b: I recommend changing the y-axis ticks to 0, 0.5, and 1.0 instead of 0.4, 0.8, and 1.2. Since the data is normalized, the 0 and 1 levels should be clearly indicated.
-Fig. 2b: The spectral data for the b-line (blue) and c-line (orange) do not seem to be displayed properly. For example, data points below -50 kHz for the b-line are missing. Please provide the complete dataset for these spectra.
-Fig. 2f: Please use a physical quantity with corresponding units (not "arb. units") for the y-axis. Such information would help readers understand signal strength.
-Fig. 2g and 2h: Please add a scale bar (e.g., 1 mm or 5 mm).
-Fig. 2i and 2j: Please add definitions for rho_ee and eta in the caption, along with an explanation of how these values were calculated. For instance, why is the b-line value almost twice the c-line value at a depth of 0 mm, even though the relative intensities of these peaks are reported as b:c = 1:0.9 on page 5? Such details would help readers better understand the discussion regarding these figures.

2.Page 6. The authors investigate reproducibility of the clock transition and state "This agreement is remarkable". Could the authors also assess how well their value agrees with the one reported in JILA's paper (C. Zhang et al. Nature 633, 63 (2024))? I imagine that determining the b-line frequency at the 10^-13 level should be relatively straightforward for the authors, given that they use a cryogenic silicon cavity at the 10^-17 level as a reference. If the values agree within 10^-13, that would be even more remarkable and should be highlighted here. If they do not agree, it would still provide critical information for future improvements in crystal fabrication.

3.Could the authors elaborate on the clock operation conditions? Specifically, what is the time constant (cut-off frequency) for the lock-in detection, and what is the integration time for the clock operation? This information would help readers better understand the instability plots in Figure 3.

4.Figure 3.

-It seems that in the S1+ phototube measurement, the instability hits a floor around 5 x 10^-13. What is the potential origin of this floor?
-Why are the data points for S1+PMT between the averaging times of 10 s and 200 s missing?
-The authors claim that “the Allan deviation follows the expected tau^-1/2 scaling till 2x10^-14”. I don’t agree with this claim. The Allan deviation for TS1+phototube clearly deviates from the 2x10^-14/tau^-1/2 line above an averaging time of 500 s, far exceeding the error bars.
-The instability of TS1+phototube deviates from the 2x10^-14/tau^-1/2 line and even shows improved instability at averaging times beyond 500 s. This behavior is counterintuitive, as instability typically worsens with longer averaging time due to drift or long-term frequency fluctuations. Could the author explain why TS1+phototube instability behaves in the opposite way here?
-Could the author clarify whether the 1/sqrt(tau) lines (dashed lines) fit to each set of instability data agree with the instability line calculated from the SNR, spectral linewidth, and carrier frequency of the nuclear transition or not? If they do not, what is the reason?
-The error bars for TS1+phototube data are difficult to see. Please use a different point type.
-What was the total measurement time for TS1 + phototube? This information is necessary for readers to evaluate the (very small) error bars at averaging times exceeding 1000 s.

5.Could the authors specify the beam size of the 148-nm laser so that the readers can estimate the excitation laser intensity inside the crystal?

6.I recommend the authors avoid using subjective words such as "remarkable" or "excellent" to describe their own results.

Referee #2

(Remarks to the Author)
This manuscript reports the operation of a nuclear clock based on the low-lying isomeric transition of 229Th embedded in a solid-state calcium-fluoride host. The authors stabilize a narrow-linewidth 148.4 nm vacuum-ultraviolet laser directly to the nuclear resonance using a fast transmission-based feedback scheme and thereby realize a functioning frequency reference derived from a nuclear rather than an electronic transition. The work demonstrates a fractional frequency instability of approximately 2×10−12/sqrt(τ) and shows agreement between independently fabricated thorium-doped crystals at the 10−13 level, providing evidence for the reproducibility of a solid-state nuclear frequency standard.

The nuclear clock is a recent hot topic, a resonant frequency for which is narrowed down to ~1 kHz level in 2024 for the first time. Multiple groups in the world are working on making a clock with this transition independently. The goal for them is the same, and unique part for this manuscript is the comparison between two crystals developed at different places. The agreement between them assures the accuracy of the clock in the future. The data presented towards Fig. 3 is reasonably and valid, but a single plot for the stability does not tell much on the implication of this achievement, and what needs to be improved for the system to be competitive compared to other state-of-the-art optical clocks.

The following questions should be answered before the manuscript is considered for publication in any journal.
Fig. 3 shows the improvement in Allan deviation when the average time gets longer, but usually, with a good enough cavity, the Allan deviation is initially flat or getting worse until the deterioration due to the cavity drift reaches to the improvement by a long enough average time. With a cryogenic Si cavity that is reported to have 4x10^-17 stability, why does the Allan deviation not have any part whose stability is dominated by that of the reference cavity?
With a stability around 10^-13, technically it is possible to perform absolute frequency measurement at the precision of ~200 Hz. Has this been performed?
How large is the error bar for TS1+phototube data on FIg. 3? If it is smaller than the size of markers, authors can consider using open circle like an Allan deviation plot on T.L. Nicholson, et al.: Nat. Commun. 6 6896 (2015).
The largest averaging time in Fig. 3 is around 3000 s. What is preventing the measurement from being longer?

The manuscript is certainly well written. However, the reviewer is afraid that the description is too technical for the broad readers of Nature. From this perspective, it is recommended to publish this manuscript on some other journals that is more oriented to technical details of a specific field.

Referee #3

(Remarks to the Author)
Review Report for the Manuscript

“A nuclear clock based on 229Th”

Submitted for publication to Nature by B. Huang et al.

The manuscript describes the development of a nuclear optical clock based on CW absorption spectroscopy of the thorium-229 nuclear clock transition.

In order to achieve this, thorium-229 doped CaF_2 crystals were grown and spectroscopically analyzed using a newly developed high-power CW laser system. The laser system was locked to a cavity-stabilized frequency comb, allowing for

absolute frequency read-out. The obtained absorption signal was used to lock the laser to the nuclear transition, thereby implementing a nuclear optical clock.

Together with a report of the Peik/Schumm collaboration, which appeared on the arXiv almost at the same time, this work marks the first development of a nuclear optical clock.
I want to congratulate the authors to this great breakthrough, which was achieved in an impressively short time.

The manuscript is well written, technically sound and fully convincing. The work marks a major breakthrough in the field of frequency metrology and is of interest to a wide circle of readers, also outside the field. For these reasons I fully recommend the publication of this work with Nature, after the following minor comments have been addressed:

1) Almost at the same time, the development of a nuclear optical clock was reported by the Peik/Schumm group (arXiv: 2606.04997). Probably due to the overlap, this paper was not referenced in the manuscript. In particular the following sentences may require revision, in view of the recent developments:

"Very recently, continuous-wave nuclear absorption spectroscopy was
achieved with sub-nanowatt 148.4 nm light and single-photon-counting photomultiplier detection [34]. However, converting nuclear laser spectroscopy into an operating clock has remained out of reach…"

I suggest to add the latest arXiv preprint to the reference list and revise the above statement accordingly.

2) The observed spectroscopy lines were interpreted based on a thorium-dimer hypothesis, as was recently suggested in the paper cited as Ref. [34]. I have the impression that this interpretation is still very much under debate and I suggest to use a more careful phrasing when discussing about the thorium dimer:

"[…] where we resolved five narrow quadrupole-split nuclear transitions associated with the Th-dimer defect centre with dihedral symmetry […]"

"The same platform resolves a broad high-symmetry O-centre transi-
tion and five narrow quadrupole components of the Th-dimer defect […]"

3) The manuscript provides a very detailed review of the work established on thorium, thereby citing practically all Nature paper that were published along this line, which is great. The more, however, I am surprised that the first direct detection of the nuclear clock transition (Nature 533, 47 (2016)) is not cited.

4) Ref. 34 was discussed in the introduction, but is also included at the end as:

"During the preparation of this work, we became aware of a report of continuous-wave nuclear absorption spectroscopy using 1 nW of 148.4 nm light from randomly
quasi-phase-matched SrB4O7 and PMT detection [34]"

In my opinion, this additional discussion is not required, since the paper was included in the introduction.

5) In Ref. [24] "CaF2" is not properly shown.

Version 1:

Reviewer comments:

Referee #1

(Remarks to the Author)
Overall, the manuscript is much improved. I still have three comments before recommending publication.

The authors have added a new dataset labeled "TS1 + phototube (optimized)". I have an impression that including only the "TS1+phototube (optimized)" data in the manuscript might be sufficient. What is the reason for including both the "TS1 + phototube" and "TS1+phototube (optimized)" datasets? Is there a physical significance to presenting both?

In the revised manuscript, the authors state that they achieved a "duty cycle of approximately 85%". Why is the 15% dead time necessary? One of the main advantages of a solid-state nuclear clock is the absence of atom preparation time (such as atom trapping or laser cooling). Therefore, providing details on why this 15% dead time is required would offer valuable insights for the future development of solid-state nuclear clocks.

Fig. 2e: The error bars are difficult to see. Please use a different point type.

Referee #2

(Remarks to the Author)
The authors responded to reviewers' comments in reasonable ways. The manuscript now looks more informative to see what their achievements are.

One concern is their way to display uncertainties.
1. Page 7 of the revised manuscript says, "an inter-crystal frequency difference of 5.6(13) ×10^2 Hz." Does the number mean (5.6 +/-0.13)x10^2 or (5.6 +/-1.3)x10^2? If latter, it should be written as 5.6(1.3)x10^2, and the reviewer believes this is the standard way to show the uncertainty. If former, one more significant digit below 5.6 should be added to avoid the confusion.
2. Same argument holds for vb,JILA = 2,020,407,298,798.58(122) kHz. Does the last three digits mean 8.58+/-1.22 or 8.58 +/- 0.122? Based on the end of Methods in p.12, The authors seem to mean the former, in which case it should be written 2,020,407,298,798.58(1.22) kHz.
3. Same comment applies to "The frequency offsets of S1 and TS1 relative to this inferred JILA value are−0.50(122) kHz and +0.06(122) kHz" in page 7 and Eq. (30).

Open Access This Peer Review File is licensed under a Creative Commons Attribution 4.0 International License, which permits use, sharing, adaptation, distribution and reproduction in any medium or format, as long as you give appropriate credit to the original author(s) and the source, provide a link to the Creative Commons license, and indicate if changes were made.
In cases where reviewers are anonymous, credit should be given to 'Anonymous Referee' and the source.
The images or other third party material in this Peer Review File are included in the article's Creative Commons license, unless indicated otherwise in a credit line to the material. If material is not included in the article's Creative Commons license and your intended use is not permitted by statutory regulation or exceeds the permitted use, you will need to obtain permission directly from the copyright holder.
To view a copy of this license, visit https://creativecommons.org/licenses/by/4.0/

Dear Dr. Levi,

Thank you for forwarding the referee reports on our manuscript "A nuclear clock based on ^{229}Th". We are grateful to the referees for their careful reading and thoughtful, constructive feedback. Their expert comments greatly improved the clarity, precision, and readability of our manuscript. We have revised the manuscript accordingly and respond point-by-point below. Referee comments are printed in blue. Our responses are in black. If changes are made to the manuscript, they are printed in red. All the citation numbers and paragraph numbers refer to the ones in the original (unrevised) manuscript unless otherwise noted.

Referee #1 (Remarks to the Author):

The manuscript "A nuclear clock based on ^{229}Th" by B. Huang et al. presents the first demonstration of a solid-state nuclear clock operation, in which the frequency of the 148-nm CW laser is stabilized to the thorium-229 nuclear transition. The error signal for the feedback loop was obtained via lock-in detection of the absorption signal. Clock operations using combinations of two home-grown Th:CaF_2 crystals and two VUV photon detectors (PMT and Phototube) were demonstrated. A clock comparison between the solid-state nuclear clock and a highly stable laser source stabilized to a cryogenic silicon cavity was also demonstrated. Frequency reproducibility among the home-grown crystals has been confirmed at the 10^{-13} level, which should provide important insights into future crystal fabrication to improve clock performance. The manuscript is well written, technically sound, and fully convincing. The achievements are worthy of publication in Nature.

However, I have significant concerns regarding the data analysis of the clock's instability. Therefore, I do not recommend the publication of the manuscript in its current form. Below, the authors will find all my concerns that would need to be considered.

Response: We thank the referee for the careful evaluation of our manuscript and for recognizing the significance of the demonstrated solid-state nuclear-clock operation and the frequency reproducibility obtained with independently grown ^{229}Th-doped crystals. We are particularly grateful for the referee's positive assessment of the technical soundness and overall quality of the work, and for the view that these achievements are worthy of publication in *Nature*.

We have taken the referee's concerns regarding the analysis of the clock instability very seriously. In response, we have carefully re-examined the instability analysis, the associated statistical uncertainties, and the interpretation of the results, and have revised the relevant figures and discussion accordingly.

Detailed responses to each concern are provided below.

1. Figure 2.

-Fig. 2b: I recommend changing the y-axis ticks to 0, 0.5, and 1.0 instead of 0.4, 0.8, and 1.2. Since the data is normalized, the 0 and 1 levels should be clearly indicated.

Response: We thank the referee for this suggestion. The y-axis ticks in Fig. 2a,b have been changed to 0, 0.5, and 1.0 so that the zero and unity levels of the normalized fluorescence signal are clearly indicated. For consistency, the y-axis ticks in Figs. 2c–e have also been changed to −1, 0, and 1, so that the zero crossing and normalized positive and negative amplitudes of the FM absorption spectroscopy signals are clearly displayed.

-Fig. 2b: The spectral data for the b-line (blue) and c-line (orange) do not seem to be displayed properly. For example, data points below -50 kHz for the b-line are missing. Please provide the complete dataset for these spectra.

Response: We thank the referee for this comment. No acquired data points were omitted from the original Fig. 2b. Rather, the fluorescence scans on S1 covered only limited frequency ranges.

Because S1 had been returned to SIOM after the original measurements and was no longer available in our setup, we repeated the fluorescence-excitation measurements over extended frequency ranges using TS1. The revised Fig. 2b now shows the complete b-line, c-line, and QU profiles (denoted as O-centre in the original manuscript) measured in TS1. For consistency, Figs. 2a and 2f have also been updated with the corresponding TS1 data.

We have also updated the figure caption and the corresponding text to identify the crystal and measurement conditions explicitly.

-Fig. 2f: Please use a physical quantity with corresponding units (not "arb. units") for the y-axis. Such information would help readers understand signal strength.

Response: We thank the referee for this suggestion. We have changed the y-axis label in Fig. 2f to "Count rate (cps)".

-Fig. 2g and 2h: Please add a scale bar (e.g., 1 mm or 5 mm).

Response: We thank the referee for this helpful suggestion. We have replaced Figs. 2g and 2h with clearer photographs of the crystals taken on grid paper and added a 5-mm scale bar to each image.

When retaking the crystal photographs, we also remeasured the crystal dimensions and activity more carefully. Based on these measurements, the diameter, length and average ^{229}Th concentration of S1 were updated from approximately 1.5 mm, 9.6 mm and 8×10^{16} cm^{-3} to 1.44 mm, 9.8 mm and 9.7×10^{16} cm^{-3}, respectively.

-Fig. 2i and 2j: Please add definitions for ρ_{ee} and eta in the caption, along with an explanation of how these values were calculated. For instance, why is the b-line value almost twice the c-line value at a depth of 0 mm, even though the relative intensities of these peaks are reported as b:c = 1:0.9 on page 5? Such details would help readers better understand the discussion regarding these figures.

Response: We thank the referee for this important comment. We agree that the definition of the quantity plotted in Figs. 2i and 2j and the procedure used to calculate it were not sufficiently clear in the original manuscript. In the revised manuscript, we have replaced ρ_{ee}/η with $p_s(z)$, defined as the depth-dependent steady-state excitation fraction associated with spectral component s. To clarify how $p_s(z)$ is calculated, we have also added a Methods subsection entitled "Resonant steady-state excitation fraction". Accordingly, we have updated Figs. 2i and 2j, and revised the corresponding caption as follows:

"**i,j**, Calculated depth-dependent steady-state excitation fraction, $p_s(z)$, for resonant excitation through line b (blue), QU (grey), and line c (orange) in S1 and TS1, respectively. Here, z is measured from the VUV entrance surface. The calculation uses the transition line strengths, measured ensemble linewidths and nuclear lifetime, together with the local VUV photon flux after accounting for surface reflection and off-resonant propagation loss through the crystal (Methods)."

We next address the referee's comparison between the calculated excitation-fraction ratio and the measured fluorescence peak ratio. Using the procedure described in Methods, we obtain

$$\frac{p_b(0)}{p_c(0)} \simeq 1.69 \tag{1}$$

for TS1. This value remains larger than the measured fluorescence peak ratio of approximately 1.2 in TS1. One possible contribution to this difference is the presence of broad pedestals associated with lines b and c, which are not included in the calculation underlying Figs. 2i and 2j (Extended Data Fig. 2).

To evaluate this contribution, we performed phenomenological double-Lorentzian fits to the full spectra of lines b and c in TS1. The fit for line b is shown in Extended Data Fig. 2. The fitted linewidths $\Delta\nu_{s,j}$ and fractional integrated spectral weights $f_{s,j}$ of the narrow components and broad pedestals are

Line	$\Delta\nu_{s,n}$ (kHz)	$\Delta\nu_{s,p}$ (kHz)	$f_{s,n}$	$f_{s,p}$
b	23.04	329.8	0.232	0.768
c	34.84	306.4	0.264	0.736

Here, $j \in \{n, p\}$, where n and p denote the narrow component and broad pedestal, respectively. Weighting the excitation fractions of the two components by their fractional integrated spectral weights gives

$$\frac{p_b^{\text{eff}}(0)}{p_c^{\text{eff}}(0)} = \frac{\chi_b \left(f_{b,n}/\Delta\nu_{b,n} + f_{b,p}/\Delta\nu_{b,p}\right)}{\chi_c \left(f_{c,n}/\Delta\nu_{c,n} + f_{c,p}/\Delta\nu_{c,p}\right)} \simeq 1.38, \tag{2}$$

where χ_s is the effective line-strength factor for spectral component s, and $p_s^{\text{eff}}(0)$ is the excitation fraction at the VUV entrance surface after accounting for both fitted spectral components. This re-evaluated value is closer to the measured fluorescence peak ratio of approximately 1.2.

2. Page 6.

The authors investigate reproducibility of the clock transition and state "This agreement is remarkable". Could the authors also assess how well their value agrees with the one reported in JILA's paper (C. Zhang et al. Nature 633, 63 (2024))? I imagine that determining the b-line frequency at the 10^{-13} level should be relatively straightforward for the authors, given that they use a cryogenic silicon cavity at the 10^{-17} level as a reference. If the values agree within 10^{-13}, that would be even more remarkable and should be highlighted here. If they do not agree, it would still provide critical information for future improvements in crystal fabrication.

Response: We thank the referee for this helpful suggestion. We have compared our absolute b-line frequencies with the JILA measurements. The frequency offsets of both S1 and TS1 relative to the inferred JILA value are consistent with zero within a combined fractional standard uncertainty of approximately 6×10^{-13}, dominated by the uncertainty of the JILA temperature-dependent fit.

Because this comparison requires the JILA frequency to be evaluated at the temperature of our crystals, we recalibrated our temperature sensor. The crystal temperature has been revised from 301 K to 301.4(1) K in the main text. We then evaluated the published JILA temperature-dependent fit at this recalibrated temperature.

We have added two Methods subsections entitled "Absolute-frequency determination" and "Inter-laboratory frequency comparison", which describe the determination of our absolute b-line frequencies and their

comparison with the inferred JILA value, respectively. The resulting comparison is summarized in the following paragraph added to the revised main text:

"For an inter-laboratory comparison, we evaluate the quadratic fit to the published JILA C10 and C13 b-line data[16] at 301.4(1) K, obtaining an inferred frequency of $\nu_{b,\mathrm{JILA}}$ = 2,020,407,298,798.58(122) kHz, where the uncertainty is a combined standard uncertainty dominated by the JILA temperature-dependent fit (Methods). The frequency offsets of S1 and TS1 relative to this inferred JILA value are −0.50(122) kHz and +0.06(122) kHz, respectively, both consistent with zero within the combined standard uncertainties. These comparisons therefore provide a quantitative assessment of the b-line frequency reproducibility across independently grown crystals and independent experimental platforms. "

3. Could the authors elaborate on the clock operation conditions? Specifically, what is the time constant (cut-off frequency) for the lock-in detection, and what is the integration time for the clock operation? This information would help readers better understand the instability plots in Figure 3.

Response: We thank the referee for suggesting that we elaborate on the clock-operation conditions. Indeed, these parameters are useful for interpreting the instability measurements in Fig. 3.

We have added Extended Data Table 1 to provide a summary of the operating conditions, which lists the modulation frequencies, modulation amplitudes, lock-in time constants, measurement times, and feedback-cycle durations for all configurations. We have also added the following sentence to the caption of Fig. 3:

"The modulation frequencies, modulation amplitudes, lock-in time constants, measurement times, and feedback-cycle durations used for the four instability measurements are summarized in Extended Data Table 1. "

4. Figure 3.

-It seems that in the S1+ phototube measurement, the instability hits a floor around 5×10^{-13}. What is the potential origin of this floor?

Response: We thank the referee for raising this point. After re-examining the instability analysis, we do not find statistically significant evidence for an instability floor in the S1 + phototube data. The apparent flattening at long averaging times remains within the corrected statistical uncertainties, which increase as the equivalent degrees of freedom decrease.

We therefore do not interpret this behaviour as a physical instability floor. In the revised Fig. 3, we have corrected the error bars and included additional measurements obtained under improved operating conditions, which show lower instability.

-Why are the data points for S1+PMT between the averaging times of 10 s and 200 s missing?

Response: We thank the referee for requesting this clarification. The data points for S1 + PMT between the averaging times of 10 s and 200 s are not missing, but unavailable owing to the sampling interval for the S1 + PMT configuration. In this configuration, one VUV frequency estimate is obtained per feedback cycle, whose duration is $T_c \simeq 225$ s. The shortest available averaging time for the Allan-deviation analysis is therefore approximately 225 s.

To make this sampling constraint explicit, we have revised the main text as follows:

" In each interrogation cycle, the normalized demodulated absorption signal is converted to a residual detuning using the calibrated discriminator slope. This detuning is fed back through an AOM in the comb-referenced optical chain (Fig. 1c) to update the VUV interrogation frequency for the next cycle. The resulting cycle-by-cycle VUV frequency record is used to calculate the in-loop fractional frequency instability shown in Fig. 3. "

-The authors claim that "the Allan deviation follows the expected $\tau^{-1/2}$ scaling till 2×10^{-14}". I don't agree with this claim. The Allan deviation for TS1 + phototube clearly deviates from the $2 \times 10^{-14}/\tau^{-1/2}$ line above an averaging time of 500 s, far exceeding the error bars.

-The instability of TS1 + phototube deviates from the $2 \times 10^{-14}/\tau^{-1/2}$ line and even shows improved instability at averaging times beyond 500 s. This behaviour is counterintuitive, as instability typically worsens with longer averaging time due to drift or long-term frequency fluctuations. Could the author explain why TS1 + phototube instability behaves in the opposite way here?

Response: We thank the referee for carefully examining the TS1 + phototube Allan deviation. We address these two related comments together.

Upon re-examining the original Fig. 3, we found that the statistical error bars had not been displayed correctly, and we apologize for this error. In the revised figure, the error bars indicate 68.3% confidence intervals calculated from the equivalent degrees of freedom of the overlapping Allan-deviation estimator under the assumed white-frequency-noise model. At long averaging times, the equivalent number of independent samples becomes small, and the Allan-deviation estimate can fluctuate above or below the underlying trend. With the corrected uncertainties, the long-averaging-time data do not show a statistically significant departure from $\tau^{-1/2}$ scaling. Nevertheless, we agree that the original claim of scaling down to 2×10^{-14} was too strong, and we do not interpret the apparent downward excursion beyond approximately 500 s as a physical improvement in clock stability. We have therefore removed this statement.

Separately, to assess the clock stability achievable under improved operating conditions, we further optimized the TS1 + phototube configuration and acquired a new clock-frequency record. The revised main text now reads:

"We then optimize the TS1 + phototube configuration by increasing the average transmitted VUV power to 350 nW, increasing the measurement duty cycle to approximately 85%, and using a near-optimal modulation amplitude of 12 kHz (Methods, Extended Data Table 1 and Extended Data Fig. 1). The overlapping Allan deviation is statistically consistent with $\tau^{-1/2}$ scaling over the measured interval, with a fitted instability of

$$\sigma_y(\tau) = 5 \times 10^{-13}/\sqrt{\tau/\mathrm{s}}.$$

"

We have also revised the Fig. 3 caption to state:

"The dashed lines show weighted $\tau^{-1/2}$ fits. Error bars indicate 68.3% confidence intervals calculated from the equivalent degrees of freedom of the overlapping Allan-deviation estimator under the assumed white-frequency-noise model."

-Could the author clarify whether the $1/\sqrt{\tau}$ lines (dashed lines) fit to each set of instability data agree

with the instability line calculated from the SNR, spectral linewidth, and carrier frequency of the nuclear transition or not? If they do not, what is the reason?

Response: We thank the referee for this important question. The dashed lines in Fig. 3 are weighted $\tau^{-1/2}$ fits to the measured overlapping Allan deviations, rather than independently calculated instability limits. Nevertheless, the fitted instabilities are consistent in magnitude with independently calculated photon-shot-noise-limited instabilities.

To make this comparison quantitative, we have added a Methods subsection entitled "Clock instability at the photon-shot-noise limit", which gives the calculation of the photon-shot-noise-limited instability and applies it to the optimized TS1 + phototube configuration.

To address the referee's question for all four measurements, we additionally apply the same calculation to the other three configurations using their corresponding experimental parameters. The calculated and measured instabilities for all four configurations are summarized below.

Configuration	Photon-shot-noise limit $\sigma_{y,\mathrm{SN}}\sqrt{\tau/\mathrm{s}}$	Measured instability $\sigma_{y,\mathrm{meas}}\sqrt{\tau/\mathrm{s}}$
S1 + PMT	1.4×10^{-11}	2×10^{-11}
S1 + phototube	7.0×10^{-12}	7×10^{-12}
TS1 + phototube	1.1×10^{-12}	2×10^{-12}
TS1 + phototube, optimized	2.9×10^{-13}	5×10^{-13}

The measured instabilities agree with the corresponding photon-shot-noise-limited values within factors of approximately 1.0–1.8, showing that photon shot noise constitutes a major contribution to the observed clock instability. The remaining differences can be partly accounted for by the gradual decrease in detected VUV power during the measurements, with residual electronic and readout noise also potentially contributing.

Finally, the SNR-based Allan-deviation expression requested by the referee, written in terms of the absorption SNR, spectral linewidth and nuclear-transition carrier frequency, is algebraically equivalent to the discriminator-slope expression given in the revised Methods.

-The error bars for TS1 + phototube data are difficult to see. Please use a different point type.

Response: We thank the referee for this helpful suggestion. In the revised Fig. 3, the TS1 + phototube data are displayed using open markers so that the error bars remain visible when they are smaller than or comparable to the marker size.

-What was the total measurement time for TS1+phototube? This information is necessary for readers to evaluate the (very small) error bars at averaging times exceeding 1000 s.

Response: We thank the referee for raising this point. The total elapsed duration was approximately 8,800 s for the initial TS1 + phototube record and 13,600 s for the optimized record added in the revised Fig. 3. The error bars in Fig. 3 have also been updated as described above, and the total record durations for all configurations are now summarized in Extended Data Table 1.

5. Could the authors specify the beam size of the 148-nm laser so that the readers can estimate the excitation laser intensity inside the crystal?

Response: We thank the referee for this suggestion. The 148.4-nm beam incident on the crystal has an

approximately Gaussian intensity profile, with an estimated $1/e^2$ intensity diameter of about 1 mm. We have added this information to the main text. The corresponding paragraph in the revised main text now reads:

"After the oven, the VUV light is spatially separated from the fundamental beams using a Brewster-angle MgF_2 prism[17] and approximately 5 μW of VUV power is delivered to the front surface of the ^{229}Th:CaF_2 crystal with a Gaussian diameter of approximately 1 mm (Fig. 1a,b)."

6. I recommend the authors avoid using subjective words such as "remarkable" or "excellent" to describe their own results.

Response: We thank the referee for this suggestion. We have removed subjective terms such as "remarkable" and "excellent" from the revised manuscript.

Referee #2 (Remarks to the Author):

This manuscript reports the operation of a nuclear clock based on the low-lying isomeric transition of ^{229}Th embedded in a solid-state calcium-fluoride host. The authors stabilize a narrow-linewidth 148.4 nm vacuum-ultraviolet laser directly to the nuclear resonance using a fast transmission-based feedback scheme and thereby realize a functioning frequency reference derived from a nuclear rather than an electronic transition. The work demonstrates a fractional frequency instability of approximately $2 \times 10^{-12}/\sqrt{\tau}$ and shows agreement between independently fabricated thorium-doped crystals at the 10^{-13} level, providing evidence for the reproducibility of a solid-state nuclear frequency standard.

Response: We thank the referee for the careful assessment of our manuscript and for the constructive comments. We appreciate the referee's acknowledgement of the nuclear-clock operation and of the reproducibility demonstrated between independently fabricated crystals.

All comments have been carefully considered, and our point-by-point responses are provided below.

The nuclear clock is a recent hot topic, a resonant frequency for which is narrowed down to ~1 kHz level in 2024 for the first time. Multiple groups in the world are working on making a clock with this transition independently. The goal for them is the same, and unique part for this manuscript is the comparison between two crystals developed at different places. The agreement between them assures the accuracy of the clock in the future. The data presented towards Fig. 3 is reasonably and valid, but a single plot for the stability does not tell much on the implication of this achievement, and what needs to be improved for the system to be competitive compared to other state-of-the-art optical clocks.

Response: We thank the referee for this thoughtful comment. We agree that the significance of this work goes beyond the instability shown in Fig. 3. The central advance is the demonstration of an operating ^{229}Th nuclear clock, in which a nuclear transition directly serves as the frequency reference. This marks a transition from precision nuclear spectroscopy to an operational nuclear frequency standard and extends time and frequency metrology from electronic to nuclear transitions.

The agreement of the clock-transition frequencies measured in independently grown ^{229}Th:CaF_2 crystals further supports the reproducibility required for a solid-state nuclear frequency reference. Future progress will depend on improving the nuclear discriminator and further reducing technical and environmental contributions to the clock instability.

Fig. 3 shows the improvement in Allan deviation when the average time gets longer, but usually, with a good enough cavity, the Allan deviation is initially flat or getting worse until the deterioration due to the cavity drift reaches to the improvement by a long enough average time. With a cryogenic Si cavity that is reported to have 4×10^{-17} stability, why does the Allan deviation not have any part whose stability is dominated by that of the reference cavity?

Response: We thank the referee for raising this point. Figure 3 shows the in-loop instability of the operating nuclear clock, rather than that of the free-running cryogenic-silicon-cavity-stabilized laser. The latter serves as a low-noise optical flywheel between nuclear interrogations. Over the relevant averaging-time range, its independently characterized instability remains below the measured nuclear-clock instability, reaching below 10^{-15} at 1,000 s. The present clock instability is therefore dominated by the nuclear-absorption measurement and feedback chain, and no cavity-dominated region is resolved.

We have clarified this point in the revised manuscript:

"The resulting cycle-by-cycle VUV frequency record is used to calculate the in-loop fractional frequency instability shown in Fig. 3. Between nuclear interrogations, the VUV laser inherits the stability of the cryogenic-silicon-cavity-stabilized optical reference through the comb-referenced optical chain. The independently characterized fractional instability of this reference remains below 10^{-15} at 1,000 s, well below the measured nuclear-clock instability at comparable averaging times[48]. This supports using the in-loop record as a faithful estimate of the instability of the operating nuclear clock."

With a stability around 10^{-13}, technically it is possible to perform absolute frequency measurement at the precision of ~200 Hz. Has this been performed?

Response: We thank the referee for raising this point. Absolute-frequency measurements of line b have been performed in both crystals, yielding statistical uncertainties well below 200 Hz and combined standard uncertainties below 200 Hz.

To report these measurements, we have revised the original main-text paragraph on the inter-crystal frequency comparison as follows:

"To test the inter-crystal reproducibility of the clock transition, we compare the centre frequencies of line b in S1 and TS1, obtaining $\nu_{b,\mathrm{S1}} = 2{,}020{,}407{,}298{,}798.077(63)_{\mathrm{stat}}(185)_{\mathrm{sys}}$ kHz and $\nu_{b,\mathrm{TS1}} = 2{,}020{,}407{,}298{,}798.635(20)_{\mathrm{stat}}(185)_{\mathrm{sys}}$ kHz, respectively (Methods). For this inter-crystal comparison, the relevant temperature contribution is set by the residual relative-temperature uncertainty. Combining this contribution with the statistical line-centre uncertainties, we obtain an inter-crystal frequency difference of $5.6(13) \times 10^2$ Hz, corresponding to a fractional frequency difference of $2.8(6) \times 10^{-13}$."

In addition, we have added a Methods subsection entitled "Absolute-frequency determination", which describes how the absolute b-line frequencies of both crystals are determined. The statistical uncertainties reported therein are obtained from the means of the complete cycle-by-cycle VUV-frequency records and can therefore be lower than those inferred from the shorter records displayed in Fig. 3.

How large is the error bar for TS1+phototube data on FIg. 3? If it is smaller than the size of markers, authors can consider using open circle like an Allan deviation plot on T.L. Nicholson, et al.: Nat. Commun. 6 6896 (2015).

Response: We thank the referee for this helpful suggestion. For much of the TS1 + phototube dataset, the error bars are smaller than or comparable to the marker size, and they become more visible at longer

averaging times. Following the referee's suggestion, we use open markers in the revised Fig. 3 so that the error bars can be seen more clearly.

We have revised Fig. 3 and its caption accordingly.

The largest averaging time in Fig. 3 is around 3000 s. What is preventing the measurement from being longer?

Response: We thank the referee for raising this point. In the revised measurement, the TS1 + phototube clock data extend to an averaging time of approximately 6,000 s. In the present setup, continuous operation is limited mainly by the long-term stability of the VUV-generation system, particularly by Cd-vapour replenishment and by Cd condensation within the vapour cell. Improved Cd-vapour management, cell geometry and thermal control should enable longer uninterrupted measurements in future implementations. We have updated Fig. 3 accordingly.

The manuscript is certainly well written. However, the reviewer is afraid that the description is too technical for the broad readers of Nature. From this perspective, it is recommended to publish this manuscript on some other journals that is more oriented to technical details of a specific field.

Response: We thank the referee for this thoughtful comment. To improve accessibility for the broad readership of *Nature*, we have substantially revised the presentation of the manuscript.

In particular, we have rewritten the "Absorption spectroscopy" and "Nuclear-clock operation and instability" sections to focus more clearly on the central physical ideas and results, while moving much of the technical detail to the Methods and Extended Data. We hope that these revisions make the manuscript more accessible while retaining the necessary technical rigour.

More broadly, we believe that the first demonstration of an operating nuclear clock represents an important step from nuclear spectroscopy to the use of a nuclear transition as an operational frequency reference, and is therefore of broad interest to the readership of *Nature*.

Referee #3 (Remarks to the Author):

Review Report for the Manuscript

"A nuclear clock based on ^{229}Th"

Submitted for publication to Nature by B. Huang et al.

The manuscript describes the development of a nuclear optical clock based on CW absorption spectroscopy of the thorium-229 nuclear clock transition.

In order to achieve this, thorium-229 doped CaF_2 crystals were grown and spectroscopically analyzed using a newly developed high-power CW laser system. The laser system was locked to a cavity-stabilized frequency comb, allowing for absolute frequency read-out. The obtained absorption signal was used to lock the laser to the nuclear transition, thereby implementing a nuclear optical clock.

Together with a report of the Peik/Schumm collaboration, which appeared on the arXiv almost at the same time, this work marks the first development of a nuclear optical clock.

I want to congratulate the authors to this great breakthrough, which was achieved in an impressively short time.

The manuscript is well written, technically sound and fully convincing. The work marks a major breakthrough in the field of frequency metrology and is of interest to a wide circle of readers, also outside the field. For these reasons I fully recommend the publication of this work with Nature, after the following minor comments have been addressed:

Response: We thank the referee for the careful evaluation of our manuscript and for the very positive and encouraging assessment of our work. We particularly appreciate the referee's recognition of the first realization of a nuclear optical clock as a major advance in frequency metrology, its broad interest beyond the immediate field, and the strong recommendation for publication in *Nature*.

We are grateful for the referee's constructive comments, which have helped us further improve the clarity and presentation of the manuscript. Below, we provide detailed point-by-point responses to each comment.

1) Almost at the same time, the development of a nuclear optical clock was reported by the Peik/Schumm group (arXiv: 2606.04997). Probably due to the overlap, this paper was not referenced in the manuscript. In particular the following sentences may require revision, in view of the recent developments:

"Very recently, continuous-wave nuclear absorption spectroscopy was achieved with sub-nanowatt 148.4 nm light and single-photon-counting photomultiplier detection [34]. However, converting nuclear laser spectroscopy into an operating clock has remained out of reach. . . "

I suggest to add the latest arXiv preprint to the reference list and revise the above statement accordingly.

Response: We thank the referee for this suggestion. We agree that the original statement should be revised in view of this independent development. We have now added the preprint by Toscani De Col et al. (arXiv:2606.04997) to the reference list and revised the corresponding text in the Introduction to acknowledge their independent and concurrent demonstration of a ^{229}Th optical nuclear clock.

The following paragraph has been added at the end of the Introduction:

"A near-simultaneous independent report has also demonstrated ^{229}Th nuclear-clock operation using the randomly quasi-phase-matched SrB_4O_7 VUV source, a highly doped ^{229}Th:CaF_2 crystal and photomultiplier-tube-based absorption readout[45], building on related continuous-wave nuclear absorption spectroscopy with sub-nanowatt 148.4 nm light[46]."

2) The observed spectroscopy lines were interpreted based on a thorium-dimer hypothesis, as was recently suggested in the paper cited as Ref. [34]. I have the impression that this interpretation is still very much under debate and I suggest to use a more careful phrasing when discussing about the thorium dimer:

"[. . .] where we resolved five narrow quadrupole-split nuclear transitions associated with the Th-dimer defect centre with dihedral symmetry [. . .]"

"The same platform resolves a broad high-symmetry O-centre transition and five narrow quadrupole components of the Th-dimer defect [. . .]"

Response: We thank the referee for this comment. We have replaced the previous O-centre and D-centre terminology with quadrupole-unresolved (QU) and quadrupole-resolved (QR), respectively.

These phenomenological labels describe the observed spectroscopic structures without implying a specific microscopic defect configuration. The corresponding terminology has been revised throughout the manuscript, including the main text, figure captions and figure labels.

3) The manuscript provides a very detailed review of the work established on thorium, thereby citing practically all Nature paper that were published along this line, which is great. The more, however, I am surprised that the first direct detection of the nuclear clock transition (Nature 533, 47 (2016)) is not cited.

Response: We thank the referee for pointing out this important omission. We agree that the first direct detection of the ^{229}Th isomer through internal-conversion decay represents a key milestone in the development of the field. We have added the work of von der Wense et al. as Ref. 8 in the revised manuscript.

The referee's comment also prompted a more comprehensive review of the reference list, and we have added and updated citations to better reflect the development of the field.

The relevant sentence in the revised Introduction now reads:

"Decades of work established the existence and energy of this low-lying state[8–11,24–30]."

4) Ref. 34 was discussed in the introduction, but is also included at the end as:

"During the preparation of this work, we became aware of a report of continuous-wave nuclear absorption spectroscopy using 1 nW of 148.4 nm light from randomly quasi-phase-matched SrB_4O_7 and PMT detection [34]"

In my opinion, this additional discussion is not required, since the paper was included in the introduction.

Response: We thank the referee for pointing this out. We have deleted this sentence in the revised manuscript.

5) In Ref. [24] "CaF_2" is not properly shown.

Response: We thank the referee for noting this formatting error. "caf_2" in Ref. [24] (now listed as Ref. [34]) has been corrected to "CaF_2".

We thank all the referees for their careful and thorough evaluation of our manuscript and for their constructive comments. The revisions prompted by their feedback have substantially improved the clarity, precision and rigour of the manuscript, and we hope that the revised version fully addresses their comments and concerns.

Dear Dr. Levi,

Thank you for forwarding the second-round referee reports on our manuscript "A nuclear clock based on ^{229}Th". We appreciate the reviewers' careful re-evaluation of the manuscript and their encouraging assessments. We have addressed each of the remaining points and made the corresponding revisions. Our point-by-point responses are provided below. Referee comments are shown in blue, our responses in black, and text added to the manuscript in red. Page, figure, and equation references in the referee comments refer to the previously revised manuscript; references in our responses refer to the present revision unless otherwise indicated.

Referee #1 (Remarks to the Author):

Overall, the manuscript is much improved. I still have three comments before recommending publication.

Response: We thank the referee for this positive assessment of our revised manuscript. We have addressed the three comments as follows.

The authors have added a new dataset labeled "TS1 + phototube (optimized)". I have an impression that including only the "TS1+phototube (optimized)" data in the manuscript might be sufficient. What is the reason for including both the "TS1 + phototube" and "TS1+phototube (optimized)" datasets? Is there a physical significance to presenting both?

Response: We thank the referee for raising this point. The two datasets are included not because they represent distinct physical regimes, but to show the stepwise improvement in clock performance. Comparing the unoptimized TS1 + phototube dataset with S1 + phototube shows the benefit of replacing the crystal while retaining the same readout. Comparing the optimized and unoptimized TS1 datasets then shows the additional gain from optimizing the VUV power, modulation amplitude and duty cycle. Presenting both therefore distinguishes the effects of crystal improvement and operating-parameter optimization.

In the revised manuscript, the authors state that they achieved a "duty cycle of approximately 85%". Why is the 15% dead time necessary? One of the main advantages of a solid-state nuclear clock is the absence of atom preparation time (such as atom trapping or laser cooling). Therefore, providing details on why this 15% dead time is required would offer valuable insights for the future development of solid-state nuclear clocks.

Response: We thank the referee for this thoughtful comment. The 15% dead time is not intrinsic to the solid-state nuclear reference, but results from data-handling and settling overhead in the present digital-feedback implementation. It could be substantially reduced through improved feedback control, allowing the duty cycle to approach unity. We have added the following clarification on page 6 of the revised manuscript:

"The remaining 15% dead time arises from technical overhead in the present digital-feedback implementation and could be substantially reduced through improved feedback control."

Fig. 2e: The error bars are difficult to see. Please use a different point type.

Response: We thank the referee for this careful observation. We have changed the data markers in Fig. 2e to open circles so that they no longer obscure the error bars. The same marker style has been applied

consistently to Figs. 2b–e.

Referee #2 (Remarks to the Author):

The authors responded to reviewers' comments in reasonable ways. The manuscript now looks more informative to see what their achievements are.

Response: We thank the referee for the positive assessment of our revised manuscript. We have addressed the remaining concerns as follows.

One concern is their way to display uncertainties.

1. Page 7 of the revised manuscript says, "an inter-crystal frequency difference of $5.6(13) \times 10^2$ Hz." Does the number mean $(5.6 + /{-0.13}) \times 10^2$ or $(5.6 + /{-1.3}) \times 10^2$? If latter, it should be written as $5.6(1.3) \times 10^2$, and the reviewer believes this is the standard way to show the uncertainty. If former, one more significant digit below 5.6 should be added to avoid the confusion.

2. Same argument holds for $\nu_{b,\mathrm{JILA}} = 2,020,407,298,798.58(122)$ kHz. Does the last three digits mean $8.58 + /{-1.22}$ or $8.58 + /{-0.122}$? Based on the end of Methods in p.12, The authors seem to mean the former, in which case it should be written $2,020,407,298,798.58(1.22)$ kHz.

3. Same comment applies to "The frequency offsets of S1 and TS1 relative to this inferred JILA value are $-0.50(122)$ kHz and $+0.06(122)$ kHz" in page 7 and Eq. (30).

Response: We thank the referee for pointing out this potential ambiguity. We respectfully clarify that our original notation follows the compact convention recognized in Section 7.2.2 of the BIPM Guide to the Expression of Uncertainty in Measurement (GUM), in which the numbers in parentheses refer to the corresponding least significant digits of the quoted value.

The intended values are $(5.6 \pm 1.3) \times 10^2$ Hz for the inter-crystal frequency difference, $2,020,407,298,798.58 \pm 1.22$ kHz for $\nu_{b,\mathrm{JILA}}$, and -0.50 ± 1.22 kHz and $+0.06 \pm 1.22$ kHz for the corresponding frequency offsets, respectively.

The alternative notation suggested by the referee, in which the absolute uncertainty is written explicitly within parentheses, is also recognized by the GUM. However, the concise final-digit convention is used consistently throughout our manuscript, including for the separately reported statistical and systematic uncertainties. We have therefore retained the original notation. The uncertainties discussed here are all 1σ combined standard uncertainties.

We believe that these revisions address all remaining comments and hope that the manuscript is now suitable for publication in *Nature*.
